# Prevalence and predictors of persistent pelvic girdle pain 12 years postpartum

**DOI:** 10.1186/s12891-017-1760-5

**Published:** 2017-09-16

**Authors:** Cecilia Bergström, Margareta Persson, Kari-Anne Nergård, Ingrid Mogren

**Affiliations:** 1 Department of Clinical Sciences, Obstetrics and Gynecology, Umeå University, Umeå, Sweden; 2 Department of Nursing, Umeå University, Umeå, Sweden; 3 Private Practice in Luleå, Luleå, Sweden

**Keywords:** Persistent pelvic girdle pain, Prevalence, Predictors, Postpartum, Long-term follow-up, Widespread pain, Sick leave, Disability pension, Cohort studies

## Abstract

**Background:**

Pelvic girdle pain (PGP) is not always a self-limiting condition. Women with more pronounced persistent PGP (PPGP) report poorer health status compared to women with less pronounced symptoms. The knowledge concerning the long-term consequences of PPGP is limited, thus more knowledge in this area is needed. The overall aim was to study the prevalence and predictors of PPGP 12 years after delivery.

**Methods:**

This is a long-term follow-up study based on a previous cohort study that commenced in 2002. New questionnaire data 12 years postpartum were collected in 2014 and early 2015. The questionnaire was distributed to a total of 624 women from the initial cohort.

**Results:**

In total, 295 women (47.3%) responded to the questionnaire where 40.3% (*n* = 119) reported pain to a various degree and 59% (*n* = 174) reported no pain. Increased duration and/or persistency of pain, self-rated health, sciatica, neck and/or thoracic spinal pain, sick leave the past 12 months, treatment sought, and prescription and/or non-prescription drugs used were all associated with an statistically significant increase in the odds of reporting pain 12 years postpartum. Widespread pain was common and median expectation of improvement score was 5 on an 11-point numeric scale (interquartile range 2–7.50). More than one of five women (21.8%) reporting pain stated that they had been on sick leave the past 12 months and nearly 11% had been granted disability pension due to PPGP. No statistically significant differences were found between respondents and non-respondents regarding most background variables.

**Conclusions:**

This study is unique as it is one of few long-term follow-up studies following women with PPGP of more than 11 years. The results show that spontaneous recovery with no recurrences is an unlikely scenario for a subgroup of women with PPGP. Persistency and/or duration of pain symptoms as well as widespread pain appear to be the strongest predictors of poor long-term outcome. Moreover, widespread pain is commonly associated with PPGP and may thus contribute to long-term sick leave and disability pension. A screening tool needs to be developed for the identification of women at risk of developing PPGP to enable early intervention.

## Background

The prevalence of persistent pelvic girdle pain (PPGP) varies among studies. However, studies have shown that women may suffer from PPGP and/or persistent low back pain 6 months to 11 years after delivery [1–6], suggesting that a spontaneous recovery without recurrences seems like an unlikely clinical course for a subgroup of women. Even though most women experience mild complaints, 13% experience moderate pain and 7% experience severe pain postpartum [7]. In addition, severity of symptom appears to vary over time [1, 8].

Most women with PPGP report a continuous dull pain, however, some women experience more intense pain sensations such as sharp and stabbing [1, 8]. We have previously reported that women with PPGP 14 months postpartum, report a higher degree of pain intensity compared to women with resolved PPGP at all prior measure points [1] and pain intensity has also been linked to a higher degree of disability in this patient group [9]. In addition, pain intensity is also closely associated to a person’s emotional wellbeing [10], and women with PPGP postpartum report a higher degree of depressive symptoms compared to women with resolved pelvic girdle pain (PGP) and/or pregnancy related low back pain [11]. Women with more pronounced symptoms postpartum also report poorer health status compared to women with less pronounced symptoms [1]. In a study investigating primiparous women’s experiences of PPGP 3 months postpartum, demonstrates that PPGP postpartum not only affect their ability to perform daily activities, i.e. lifting and household activities, but also slow them down and increase their worrying about the progression of their symptoms [8].

Low back pain (LBP) is considered to be the number one leading cause of years lived with disability globally within the musculoskeletal condition group [12]. The estimated cost due to chronic musculoskeletal conditions in Sweden is estimated at Swedish krona (SEK) 87.5 billion in 2006, where over 90% constitutes indirect costs such as sickness absence and disability pension [13]. It has been suggested that women with PPGP may constitute a specific subgroup of patients within the heterogeneous back pain population making their long-term outcome less favourable [1].

At present, knowledge of the long-term outcome for women with PPGP is limited. To date, there are relatively few studies concerning PPGP after pregnancy with a follow-up of 11 years or more [2]. Since the long-term outcome of PPGP appears to be poor according to available evidence and that it constitutes a significant health problem for many women, more knowledge is needed.

## Aim

The overall aim was to study the prevalence of PPGP 12 years after delivery in women reporting pain during their pregnancy in 2002. More specifically, we wanted to describe the women by various background variables as well as compare differences in women reporting pain versus no pain and identify variables associated with PPGP 12 years postpartum.

## Methods

### Study design

**Fig. 1:**
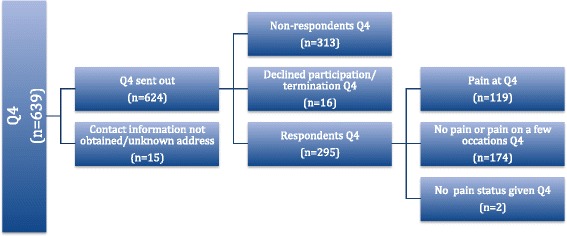
Flowchart of the cohort

This study is a long-term follow-up study based on a previous cohort study (*N* = 639) where the primary data collection took place between 2002 and 2003 through a series of three questionnaires (Q) distributed to patients: 1) in close proximity to delivery in 2002 (Q1), 2) 6 months postpartum (Q2), and 3) 14 months postpartum (Q3). Details of the primary cohort are described in detail elsewhere [1, 14]. In this study, new questionnaire data (Q4) were collected in 2014 and early 2015. Figure 1 gives a visual overview of the present follow-up cohort through a flowchart.

### Data collection

The fourth questionnaire was distributed to all participants who reported PGP at Q1 (*N* = 639). Current addresses to the eligible participants were obtained from Statens personadressregister (SPAR). SPAR includes all persons registered as residents in Sweden and is updated each day with data from the Swedish Population Register which includes all inhabitants in Sweden.

The questionnaire (Q4) was marked with a unique identification number. Date of distribution and of reminders, informed consent (IC) received, and completion of the questionnaire were registered on a spreadsheet containing contact information. The spreadsheet was also used to enter information regarding declining to participate in the study as well as how this information was received (e-mail or phone call). Three individuals carried out the distribution process (CB, MP, and an administrator). The first invitations to participate in the study were sent to eligible participants between May and June in 2014. A first reminder was sent after 3 to 5 months. A second reminder was sent 6 to 7 months after the initial distribution and a final third reminder was distributed 8 to 10 months after the first invitation. During the reminder process where new questionnaires were sent to eligible participants, it was noted that 37 individuals who already had responded to the questionnaire had answered the Q4 a second time.

Questions in Q4 were similar to questions posed in Q1-Q3 to enable comparisons over time [1, 4, 14]. Consequently, Q4 investigated different health outcomes after delivery in 2002 [14]. Additionally, validated instruments such as the EQ-5D (health status, health profile and quality of life) [15, 16] Roland Morris Disability Questionnaire (RMDQ) [17] and the Swedish version of the Multidimensional Pain Inventory (MPI-S) [18] were included in Q4 as these instruments have been shown to work well for patient with chronic musculoskeletal pain. However, results from these instruments will be presented in subsequent articles.

### Study participants

Current addresses of a total of 624 of 639 of eligible participants (97.4%) were successfully obtained from the Swedish Population Register (SPAR). Three had changed their addresses when the Q4 was sent out, rendering no response. Written informed consent was obtained from 295 participants rendering a total response rate of 47.3% (Fig. 1).

### Definitions of variables

In consensus with the previous definitions in questionnaire Q1-Q3, *PPGP* was defined as ‘continuous’ or ‘recurrent’ LBP or pain in the pelvic area over the past 12 months. Response alternatives to the question ‘In the past 12 months, have you had pain in your low back and/or pelvis?’ were ‘yes, continuous pain’, ‘yes, recurrent pain’, ‘yes, pain on a few occasions’, and ‘no pain’. The response alternative were in concordance with previous questionnaires with the exception of ‘yes, pain on a few occasions’ that was added in Q4. In addition, women who reported ‘continuous’ and ‘recurrent’ pain were also asked to mark the area of pain on a schematic drawing, which has been described previously [14], in the questionnaire.

*Mean age* was calculated by subtracting the date of birth from January 1, 2015 as not all responders had given a specific date to when the Q4 was filled out.

*Sciatica* was defined as pain in the leg or both legs in connection with LBP/pelvic pain the past 12 months with the response alterative ‘yes’ or ‘no’.

#### Neck pain (NP) and/or thoracic spinal pain (TSP)

Participants were asked if they had had ‘pain in their neck or between the shoulder blades the past 12 months’ with the response alternative ‘yes, continuous pain’, ‘yes, recurrent pain’, ‘yes, pain on a few occasions’, and ‘no pain’. No schematic drawing was provided to mark the area of pain.

*Days of pain* were measured by asking the participants ‘how many days in the past 12 months in total they have had ‘LBP/pelvic pain’ with response alternatives ‘less than 30 days’ and ‘more than 30 days’ and the same was done in regard to NP/TSP.

### Health and lifestyle questions

*Healthcare services* were defined as healthcare/treatment provided by a practitioner in the allopathic medicine or complementary and alternative medicine (CAM). Participants were asked if they had sought healthcare due to LBP/PGP after their last pregnancy according to their obstetric history and what kind of care they had sought. It was possible to give more than one option. In addition, questions were asked in regard to perceived effect of a specific treatment sought with response alternatives ‘no effect’, ‘some effect’, and ‘good effect’.

#### Expectation outcomes

The women were asked to rate their chance of getting substantially better on an 11-point numeric rating scale (NRS) where 0 denotes ‘no chance’ and 10 ‘very good chance’.

#### Sick leave and disability pension

The participants were asked if they had been on sick leave due to LBP and/or pelvic pain in the past 12 months and if so how many days with the options ‘1–7 days in total’, ‘8–14 in total’, and ‘more than 15 days in total’. In addition, they were asked to what degree they had been on sick leave with the option ‘full-time’ and ‘part-time (including to what degree in percentage)’. Granted disability pension was investigated through the question ‘have you been granted disability pension due to LBP and/or pelvic pain’ with response alternatives ‘yes’ and ‘no’.

*Physical activity* was reported through the question: Have you exercised/undertaken sports on a regular basis since your last pregnancy? The response alternatives were ‘yes’ or ‘no’.

#### Body mass index (BMI)

Current self-reported weight at Q4 was asked for in kilograms (kg) and the height was given in centimetres (cm) and extracted from Q1. Current BMI was defined as kilograms (kg)/height^2^ (meters). The WHO classification principal cut-off points were used for adult underweight, normal range, overweight, and obesity: i.e. underweight <18.50 kg/m^2^, normal range 18.50–24.99 kg/m^2^, overweight ≥25.00 kg/m^2^, and obesity ≥30.00 kg/m^2^.

#### Self-rated health status (SRH)

The women were asked to assess their current overall health status through a five category response alternative with the options: ‘very good’, ‘quite good’, ‘fair’, ‘quite poor’, and ‘poor’.

*Tobacco use* was investigated by asking the women if they smoked or used snuff as well as the amount of cigarettes smoked per day and snuffboxes used per week.

The use of *alcohol consumption* was measured by using one of the standard questions in the questionnaire Alcohol Use Disorders Identification Test (AUDIT); ‘how often do you drink alcohol?’ with the response alternatives ‘never’, ‘once a month or less’, ‘2–4 times a month’, ‘2–3 times a week’, and ‘4 times a week or more often’ [19].

### Relationship satisfaction questions

#### Family situation

The options available regarding current civil state were ‘married’, ‘cohabiting’, ‘in a relationship but not cohabiting’, and ‘single’. The women were also asked if they had changed partners since their last pregnancy in 2002 (response alternative ‘yes’ or ‘no’) and how they perceived their current relationship by the response alternatives through the question: ‘How do you perceive the relationship between you and your partner?’ with the response alternatives: ‘very good’, ‘good’, ‘neither good nor bad’, ‘bad’, and ‘very bad’. In addition, women were asked if the relationship with their current partner had changed since their last pregnancy with the response alternatives ‘improved’, ‘worsened’, ‘no change’ and ‘don’t know’.

*Satisfaction with sexual life* were investigated by asking the women if they were satisfied with their sexual life after their last pregnancy (‘yes’, ‘no’, ‘don’t know’, and ‘don’t have a sexual life at the moment’) and if their sexual life had changed since their last pregnancy (‘improved’, ‘worsened’, ‘no change’ and ‘don’t know’).

### Statistical methods

To investigate the prevalence of PPGP 12 years postpartum as well as other background variables descriptive statistics was used. Data were analysed through calculation of means and standard deviations (SD) for parametric data. Independent-samples t-test and Pearson’s Chi-square test was used to test for difference between respondents and non-respondents, as applicable, on variables collected at Q1. To test for differences between women reporting pain at Q4 compared to women reporting ‘no pain’ the independent-sample t-test and Pearson’s Chi-square test was used as appropriate. Univariate logistic regression was used to calculate the odds ratio (OR) for reporting pain 12 years postpartum (Q4) using the ‘no pain’ group as the predefined reference group. All variables from the univariate analyses with a *p*-value of 0.20 or less were used in the stepwise backward multivariate logistic regression using the likelihood ratio criteria. In addition, we used the Cronbach’s alpha to evaluate the internal response consistency in the 37 duplicate questionnaires received by the women who had completed the Q4 twice. Statistical significance was set at *p* < 0.05 for all analyses. IBM SPSS Statistics 24 software package was used.

## Results

The mean maternal age of all respondents at Q1 was 30.7 years and mean age at filling out Q4 was 42.9 years, yielding a mean time distance of 12.1 years (SD and 95% confidence interval (CI) in parenthesis) between Q1 and Q4 (SD 0.3, 95% CI 12.1–12.1). Out of the total respondents (*N* = 295), *n* = 119 (40.3%) reported pain to a various degree, and *n* = 174 (59.0%) reported ‘no pain’ or ‘pain on a few occasions’ and thus were placed in the ‘no pain’ group. One woman reported to be pregnant when responding to Q4. Relationship satisfaction to the partner since the last delivery was rated as ‘good’ or ‘very good’ by most women and few reported that their relationship had changed positively or negatively since Q1. In general, the women were satisfied with their sexual life and did not feel that it had changed since Q1.

Table 1 describes the study sample and the characteristics of women at baseline at Q4 with a mean age of 43.3 years (SD 4.6). Almost 90% of the women were married or cohabiting and most had not changed partner since 2002. The mean number of children born after 2002 (Q1) was 1.5 with 95% CI 1.3–1.6 (median 1 child). Almost 50% of the women reported to be of ‘normal weight’ and the mean BMI (kg/m^2^) was 25.6 (SD 5.2) at Q4. Women with PPGP at Q4 reported their SRH to be ‘fair to poor’ to a statistically significant higher degree compared to women reporting ‘no pain’ (*p* < 0.0001). Most women (69.9%) reported that they participated in regular physical activity.

**Table 1:** Descriptive information and comparison between women reporting pain versus no pain 12 years postpartum

	Study group	No pain	Pain	*P*-value*
	*n = 295*	*n = 174*	*n = 119*	
Age in years, mean (SD)	43.3 (4.6)	43.5 (4.4)	42.9 (4.9)	0.14
Marital status				
Married/cohabiting	223 (87.8)	130 (88.4)	92 (87.6)	0.43
Relationship but not cohabiting	9 (3.5)	3 (2.0)	5 (4.8)	
Single	22 (8.7)	14 (8.0)	8 (7.6)	
Educational level at Q1				
Up to high school/folk school	151 (52.2)	86 (50.9)	64 (54.2)	0.58
University or higher	138 (47.8)	83 (49.1)	54 (45.8)	
Number of children born after 2002				
1	101 (65.2)	64 (66.7)	36 (62.1.)	0.84
2	44 (28.4)	26 (27.1)	18 (31.0)	
≥ 3	10 (3.4)	6 (6.3)	4 (6.9)	
Total number of children				
1	70 (23.7)	41 (23.6)	28 (23.5)	0.85
2	86 (52.9)	49 (28.2)	37 (31.1)	
≥ 3	139 (47.1)	84 (48.3)	54 (45.4)	
BMI				
Normal weight	142 (49.5)	84 (49.4)	57 (49.6)	0.98
Obesity, overweight, underweight	145 (50.5)	86 (50.6)	58 (50.4)	
Low back pain before pregnancy in 2002				
No	55 (52.9)	28 (58.3)	27 (48.2)	0.12
Yes	49 (47.1)	20 (41.7)	29 (51.8)	
Self-rated health the past 12 months				
Quite good to very good	187 (63.8)	134 (77.5)	52 (44.1)	<0.0001
Fair to poor	106 (36.2)	39 (22.5)	66 (55.9)	
Physical activity				
Yes	204 (69.9)	127 (73.4)	76 (65.0)	0.12
No	88 (30.1)	46 (26.6)	41 (35.0)	
Days with PPGP^a^ the past 12 months				
< 30 days	89 (44.7)	67 (83.8)	21 (17.9)	<0.0001
≥ 30 days	110 (55.3)	13 (16.3)	96 (82.1)	
Sciatica				
No	79 (39.9)	46 (57.5)	33 (28.4)	<0.0001
Yes	119 (60.1)	34 (42.5)	83 (71.6)	
Neck or thoracic pain the past 12 months				
No	91 (45.5)	48 (59.3)	43 (36.8)	0.002
Yes	109 (54.5)	33 (40.7)	74 (63.2)	
Days with neck or thoracic pain the past 12 months				
< 30 days	62 (37.1)	31 (47.7)	31 (31.0)	0.03
≥ 30 days	105 (62.9)	34 (52.3)	69 (69.0)	
Sick leave due to PPGP^a^ the past 12 months				
No	167 (86.5)	71 (93.4)	94 (81.7)	0.05
Yes	26 (13.5)	5 (6.6)	21 (18.3)	
Days on sick leave due to LBP^2^/PPGP^1^				
< 15 days	13 (48.1)	5 (83.3)	8 (38.1)	0.05
≥ 15 days	14 (51.9)	1 (16.7)	13 (61.9)	
Degree of sick leave due to LBP^b^/PPGP^a^				
Fulltime	24 (88.9)	4 (100.0)	20 (87.0)	0.44
Part time	3 (11.1)	–	3 (13.0)	
Disability pension due to PPGP^a^				
No	177 (93.2)	74 (97.4)	101 (90.2)	0.06
Yes	13 (6.8)	2 (2.6)	11 (9.8)	
Treatment sought since last delivery due to PPGP^a^				
No	106 (53.8)	54 (67.5)	50 (43.5)	0.001
Yes	91 (46.2)	26 (32.5)	65 (56.5)	
Prescription and/or non-prescription drugs^c^				
No	160 (54.8)	109 (63.0)	51 (43.6)	0.001
Yes	132 (45.2)	64 (37.0)	66 (56.4)	
Smoking				
No	280 (94.9)	168 (96.6)	111 (93.3)	0.20
Yes	15 (5.1)	6 (3.4)	8 (6.7)	
Snuff				
No	269 (91.2)	159 (91.4)	108 (90.8)	0.85
Yes	26 (8.8)	15 (8.6)	11 (9.2)	
Alcohol consumption (AUDIT^d^)				
Never	41 (13.9)	22 (12.6)	18 (15.1)	0.43
Once a month or less	110 (37.3)	59 (33.9)	50 (42.0)	
2–4 times a month	120 (40.7)	78 (44.8)	42 (35.3)	
2–3 times a week	23 (7.8)	14 (8.0)	9 (7.6)	
4 times a week or more often	1 (0.3)	1 (0.6)	–	

Background descriptive information of participants and comparison for difference between women reporting pain versus no pain at Q4 unless otherwise specified (analysed by Pearson’s chi-square and t-test as appropriate)

Numbers in parenthesis are percentage unless otherwise specified

^a^ Persistent pelvic girdle pain (PPGP)

^b^ Low back pain (LBP)

^c^ Use of prescription and/or non-prescriptions drugs on a regular basis

^d^ Alcohol Use Disorders Identification Test (AUDIT)

* Significance test *p* < 0.05

Over 55% (*n* = 110) reported PPGP of ≥30 days the past 12 months and reported PPGP at Q4 to a higher extent compared to women with ‘no pain’ (*p* < 0.0001). Women with pain at Q4 more often reported NP and/or TSP the past 12 months (*p* = 0.002) and sciatica (*p* < 0.0001) compared to women reporting ‘no pain’. In total, 26 women (13.5%) reported having been on sick leave due to PPGP and/or LBP in the past 12 months demonstrating a significant difference between women reporting ‘pain’ versus ‘no pain’ (*p* = 0.05). Furthermore, one of five women (21.8%) reporting ‘pain’ at Q4 reported that they had been on sick leave in the past 12 months and most had been so on a full-time basis with duration of more than 15 days. In addition, a total of 13 women (6.8%) had been granted disability pension due to PPGP at Q4 with the majority belonging to the ‘pain’ group.

**Fig. 2:**
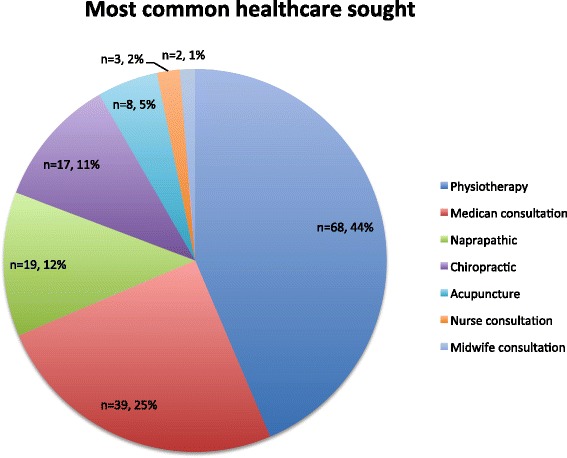
Most common healthcare sought the past 12 months at Q4

**Fig. 3:**
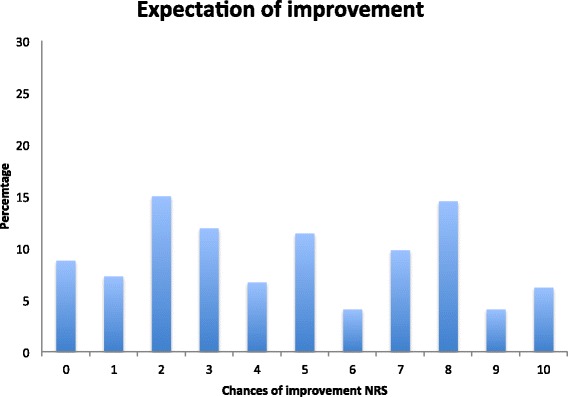
Reported expectation of improvement using 11-point numeric rating scale (NRS) where 0 denotes ‘no chance’ and 10 ‘very good chance’

More than half of the women (53.8%) had not sought any healthcare due to PPGP since their last delivery. However, women with reported pain at Q4 has sought treatment and used prescription and/or non-prescription drugs to a higher extent compared to women reporting ‘no pain’ (*p* < 0.0001 and *p* < 0.0001 respectively). For those who had sought healthcare due to PPGP in the past 12 months, physiotherapy was the most common service pursued followed by consultation with a medical doctor (Fig. 2). A minority of women smoked or used snuff (5.1% and 8.8% respectively). The most commonly reported alcohol intake was 2–4 times a month (40.7%). The responding women had a median expectation of improvement score of 5 (interquartile range [IQR], 2–7.50) (Fig. 3).

Table 2 show that women assessing their SRH as ‘fair to poor’ the past 12 months were more than four times as likely (with 95% CI in parenthesis) to report pain at Q4 compared to women reporting ‘quite good to very good’ SRH (OR 4.36, (2.62–7.26), *p* < 0.0001). Further, the OR of reporting pain at Q4 for women reporting sciatica or NP/TSP the past 12 months was: OR 3.40 (1.87–6.20), *p* < 0.0001, and OR 2.50 (1.40–4.48), *p* = 0.002, respectively. Women reporting ≥30 days with PPGP the past 12 months were 23 times as likely to report pain at Q4 compared to women reporting no pain, whereas NP/TSP for ≥30 days the past 12 months was associated with an increased OR of 2.03 (1.06–3.87), *p* = 0.03. Sick leave the past 12 months was associated with a three-fold increase in the OR of reporting pain at Q4. Additionally, there was a two- to three-fold increase in the likelihood of reporting pain at Q4 if treatment had been sought since the last delivery and if prescription and/or non-prescription drugs were used on a regular basis.

**Table 2:** Odds ratio for reporting pain 12 years postpartum using univariate logistic regression

Pain or no pain 12 years postpartum	No pain	Pain	Univariate OR for pain at Q4	95% CI^1^	*p*-value*
	*n = 174*	*n = 119*			
Age in years, mean (SD)	43.5 (4.4)	42.9 (4.9)	0.97	0.92–1.02	0.27
Low back pain before pregnancy in 2002					
No	20 (41.7)	29 (51.8)	1		
Yes	28 (58.3)	27 (48.2)	1.05	0.62–1.80	0.86
Self-rated health the past 12 months					
Quite good to very good	134 (77.5)	52 (44.1)	1		
Fair to poor	39 (22.5)	66 (55.9)	4.36	2.62–7.26	<0.0001
Days with PPGP^a^ the past 12 months					
< 30 days	67 (83.8)	21 (17.9)	1		
≥ 30 days	13 (16.3)	96 (82.1)	23.56	11.03–50.32	<0.0001
Sciatica					
No	46 (57.5)	33 (28.4)	1		
Yes	34 (42.5)	83 (71.6)	3.40	1.87–6.20	<0.0001
Neck or thoracic pain the past 12 months					
No	48 (59.3)	43 (36.8)	1		
Yes	33 (40.7)	74 (63.2)	2.50	1.40–4.48	0.002
Days with neck or thoracic pain the past 12 months					
< 30 days	31 (47.7)	31 (31.0)	1		
≥ 30 days	34 (52.3)	69 (69.0)	2.03	1.06–3.87	0.03
Sick leave due to PPGP^a^ the past 12 months					
No	71 (93.4)	94 (81.7)	1		
Yes	5 (6.6)	21 (18.3)	3.17	1.14–8.82	0.03
Days on sick leave due to LBP^b^/PPGP^a^					
< 15 days	5 (83.3)	8 (38.1)	1		
≥ 15 days	1 (16.7)	13 (61.9)	8.13	0.80–82.73	0.08
Disability pension due to PPGP^a^					
No	74 (97.4)	101 (90.2)	1		
Yes	2 (2.6)	11 (9.8)	4.03	0.87–18.73	0.08
Treatment sought since last delivery due to PPGP^a^					
No	26 (32.5)	65 (56.5)	1		
Yes	54 (67.5)	50 (43.5)	2.7	1.49–4.90	0.001
Prescription and/or non-prescription drugs^c^					
No	109 (63.0)	51 (43.6)	1		
Yes	64 (37.0)	66 (56.4)	2.20	1.37–3.56	0.001

Odds ratio (OR) and their 95% confidence intervals (CI) for reporting pain 12 years postpartum in women reporting pain during pregnancy in 2002 analysed by univariate logistic regression

^a^ Persistent pelvic girdle pain (PPGP)

^b^ Low back pain (LBP)

^c^ Use of prescription and/or non-prescriptions drugs on a regular basis

Numbers in parenthesis are percentage unless otherwise specified

^1^ 95% confidence interval

* Significance test *p* < 0.05

The 5th and final stepwise backward multivariate logistic regression model included days of PPGP the past 12 months, sciatica, NP/TSP the past 12 months, and days with NP/TSP the past 12 months and rendered in a statistically significant model with a Hosmer-Lemeshow test result of χ^2^ = 6.93, 6 degrees of freedom, *p* = 0.33 indicating a satisfactory goodness of fit (Table 3). Nevertheless, the Nagelkerke *R* ^*2*^ of 0.510 indicates that the model by itself is poor in predicting pain 12 years postpartum. In other words, the explanatory variables statistically significantly contribute to the prediction of the model, however the effect size is small.

**Table 3:** Odds ratios of reporting pain 12 years postpartum using multivariate logistic regression

Pain or no pain 12 years postpartum	No pain	Pain	Multivariate OR for pain at Q4	95% CI^1^	*p*-value*
	*n = 174*	*n = 119*			
Days with PPGP^a^ the past 12 months					
< 30 days	67 (83.8)	21 (17.9)	1		
≥ 30 days	13 (16.3)	96 (82.1)	23.08	9.10–58.53	<0.0001
Sciatica					
No	34 (42.5)	83 (71.6)	1		
Yes	26.4 (57.5)	33 (28.4)	2.31	0.93–5.73	0.07
Neck or thoracic pain the past 12 months					
No	48 (59.3)	43 (36.8)	1		
Yes	33 (40.7)	74 (63.2)	0.21	0.05–0.86	0.03
Days with neck or thoracic pain the past 12 months					
< 30 days	31 (47.7)	31 (31.0)	1		
≥ 30 days	34 (52.3)	69 (69.0)	4.61	1.27–16.75	0.02

Odds ratio (OR) and their 95% confidence intervals (CI) for reporting pain 12 years postpartum in women reporting pain during pregnancy in 2002 using multivariate logistic regression

^a^ Persistent pelvic girdle pain (PPGP)

Variables with a *p*-value of 0.20 or less in the univariate analysis of life style and health status respectively were entered in a stepwise backward manner using the likelihood criteria. Only the final model of the multivariate regression analysis is presented

Numbers in parenthesis are percentage unless otherwise specified

^1^ 95% confidence interval

* Significance test *p* < 0.05

### Non-respondents and consistency analysis

Almost all background variables collected at Q1 showed no statistically significant differences between the respondents and non-respondents with the exception that non-respondents had a higher number of children and had undergone a higher number of pregnancies at Q1 compared to the respondents. In addition, statistically significant differences were found in regard to age at Q4, where respondents were slightly older (mean age 43.3 years, SD 4.6) compared to non-respondents (mean age 42.3 years, SD 4.9), *p* = 0.013. Non-respondents reported a higher alcohol intake 3 months before the pregnancy in 2002 (Q1) compared to respondents (*p* = 0.04). Marital status also demonstrated significant differences between respondents and non-respondents, where non-respondents were single or in a relationship but not cohabiting to a higher extent compared to respondents (*p* = 0.04). However, the difference between the groups disappeared when ‘married’ and ‘cohabiting’ was merged.

The mean time distance from the first response of Q4 to the second response was calculated to be 6.5 months (SD 1.8, 95% CI 5.9–7.1). Background variables such as age, education level, marital status, number of children, and SRH at Q4 showed no statistically significant differences between those who responded once compared to those who responded twice. Consistency analysis of the 37 duplicate questionnaires received showed an excellent agreement (Cronbach’s alpha α ≥ 0.9) regarding prevalence of PPGP the past 12 months, weight, oral contraceptives (OC) use, smoking, alcohol consumption, regular use of prescription and non-prescription drugs, marital status, and change of partner. Good agreement (Cronbach’s alpha 0.9 > α ≥ 0.8) was demonstrated for pain intensity the past 12 months, neck and/or thoracic pain the past 12 months, NP and/or TSP for more or less than 30 days, physical exercise in the past 6 months, healthcare sought in the past 12 months, effect of chiropractic care, relationship satisfaction, sex life satisfaction. Further, LBP more or less than 30 days, sciatica in the past 12 months, pain intensity during the past week, physical activity between pregnancies, SRH, effect of massage treatment, and change in regard to relationship satisfaction showed acceptable agreement (Cronbach’s alpha 0.8 > α ≥ 0.7). However, questionable agreement (Cronbach’s alpha 0.7 > α ≥ 0.6) was shown for PGP during pregnancy of second child born after 2002, expectation of improvement and effect of analgesic. In addition, poor agreement (Cronbach’s alpha 0.6 > α ≥ 0.5) changes in satisfaction of sexual life after last delivery and unacceptable agreement (Cronbach’s alpha 0.5 > α) regarding PGP during the pregnancy of first child born after 2002 and physical exercise since last delivery.

## Discussion

The main purpose of this study was to determine the prevalence and predictors of PPGP 12 years postpartum. To the best of the authors’ knowledge, this is one of the few long-term follow-up studies of more than 11 year concerning the prevalence of PPGP in women developing PGP during pregnancy [2]. Our results demonstrate that about 19% of women (*n* = 119) reported pain to a various degree 12 years postpartum. These results are similar in comparison to other long-term follow-up studies of 3 and 6 years [20, 21]. In addition, women reporting PPGP for ≥30 days in the past 12 months were 23 times more likely to report pain at Q4 compared to women reporting <30 days of pain. Thus confirming previous findings showing that duration and/or persistence of pain are strong predictors of poor health outcome [22–24] and a poor chance of long-term recovery [25].

This study demonstrated that more than 1 in 5 women reporting pain at Q4 had been on sick leave in the past 12 months and most had been so on a full-time basis. In addition, women with reported sick leave the past 12 months were twice as likely to report pain at Q4. Several studies demonstrate that previous sick leave increases the risk of subsequent episodes of sickness absence [26–30], where numbers of preceding spells of sick leave increase the risk of future sick leave [27]. Musculoskeletal conditions have been observed to show high recurrences of sick leave [28, 29], where short absence spells are more common in women than in men and longer spells of sick leave in women are due to poor health [31]. In addition, there is a strong association between the risk of receiving disability pension and previous amount of annual sick days and long-term absence spells [32], which may partly explain the results in this study where nearly 11% of women with pain at Q4 had been granted disability pension due to their PPGP. The results in this study further confirm previous research demonstrating that PGP is not a self-limiting condition for a subgroup of women [1, 2, 20]. Instead, it appears that PGP symptoms, developed during pregnancy, may progress into a more chronic condition with negative long-term socioeconomic consequences, production loss, and rehabilitation expenditures.

Neck pain is the second most common musculoskeletal disorder only preceded by LBP. It has been estimated that the lifetime prevalence of NP is 50% and the one-year prevalence has been estimated to 30 to 50% [33, 34]. Even though less prevalent than LBP and NP, TSP is more common in women than in men [35]. In fact, 1 in 5 women suffer from TSP compared to 1 in 10 men [35]. Emerging evidence reveals that women with regional spinal pain in particular are more susceptible to develop chronic widespread pain [36]. It has been further suggested that women with chronic LBP should be evaluated for widespread pain [37]. Moreover, multiple pain sites and longer duration of pain have also been shown to be prognostic factors associated with disability in patients with LBP [38]. In our study, current work description was not available making it impossible to draw any conclusion whether or not the self-reported NP and/or TSP was work-related. Nevertheless, women appear to report more upper body musculoskeletal issues as well as more severe symptoms compared to men [39]. This study demonstrated that most women did not only report multiple pain sites concomitant with PPGP (i.e. sciatica and/or NP/TSP) but also pain duration of ≥30 days, all which were associated with an increased OR (ranging between 2 and 3.4) of reporting pain at Q4. These results suggest that some women with PPGP may develop widespread pain patterns, hence having a poorer prognosis.

A strong personal belief, that back pain will be of a long lasting nature, is a strong clinically significant factor predicting both short-term and long-term outcomes [40]. Measurement of recovery expectation using a specific, time-based measure within 3 weeks of non-specific LBP has been shown to be a strong predictor of poor health outcome [41]. Individuals not expecting a short-term recovery have been demonstrated to accurately predict their outcome [41]. However, it is not clear whether it is a “…self-fulfilling prophecy or a correct identification of obstacles to their own recovery” [41]. When symptoms become more chronic in nature, recovery expectation measurement tend to provide a weaker prediction of outcome [41] and/or it may be that recovery expectations are more related to previous experiences with LBP than to the severity of symptoms [42]. Due to the chronic nature of PPGP in some women it is not surprising that most women in this study reported a low expected improvement of their PPGP symptoms.

The main reason why patients with LBP seek care is decreased physical functioning as well as a desire to find the cause of pain [43]. More than half of the women in this study had not sought care for their pain problems and could possibly be explained that almost 70% women were physically active, suggesting that they did not experience a reduced physical functioning. However, it has been proposed that women with PPGP may feel neglected by healthcare professionals when bringing up their symptoms [44], which may explain why just over 50% of women reporting pain at Q4 had sought healthcare for their PPGP symptoms since their last pregnancy. Furthermore, to date there are no proven effective treatments for this condition [45–47], particularly when PGP have transitioned into chronicity. Nonetheless, women were 2–3 times as likely to report pain at Q4 if treatment had been sought since last delivery and if they used prescription and/or non-prescription drugs on a regular basis.

Most women in this study could be perceived as fairly healthy individuals as nearly 70% took part in physical activity and about half of the women were considered to be of normal weight. In addition, very few women reported to use tobacco on a regular basis and the majority reported a moderate intake of alcohol. Nevertheless, LBP has been found to contribute to poor SRH [48] and in this study we could demonstrate that more than 55% of women reporting pain at Q4 also rated their health to be ‘fair to poor’. Additionally, even though SRH did not add any predictive value in the multivariate logistic regression model, reporting ‘fair to poor’ SRH increased the odds of reporting pain at Q4 with more than 4 times. These result somewhat contradict previous results from the same cohort demonstrating where most women rated their health status as ‘quite good’ to ‘very good’ during pregnancy, 6 months after pregnancy and 14 months after pregnancy [1]. On the other hand, prolonged pain issues may lessen the total experience of health. Marriage satisfaction has shown to be particularly beneficial in regard to health and welfare [49], where there appear to be a positive association between marriage and good health status [49]. In our study, the relationship satisfaction was rated high by respondents and appeared to be quite stable over time. In addition, most women were satisfied with their sexual life. Yet, almost 18% of the women reported that they had changed partner since their pregnancy in 2002.

### Methodological considerations

There are some limitations of this study that need to be discussed. Generally, a response rate of 50% is considered adequate for analysis and reporting, while 60% is considered as ‘good’ [50]. Response rate seem to vary depending on what kind of survey is being conducted as well as to whom the survey is directed to [50]. A high non-response rate affects the quality of data by reduced sample size and could potentially introduce bias if non-respondents differs from respondents [51]. In this study we did not reach a response rate of more than 50% despite several reminders. Moreover, the questionnaire used in this study consisted of 106 questions and several studies have demonstrated that longer questionnaires seem to negatively affect the response rate [52–55]. Currently, there is insufficient evidence regarding the optimal length (in terms of number of pages or questions). However, the odds of response of one single page have been demonstrated to be twice than that with three pages [52]. Nevertheless, it has been shown that it is possible to achieve a response rate of more than 60% even for long-term follow-up studies of 12 years [56]. Others have suggested that more attention should be devoted to assessment of bias instead of a specific response rate threshold [57]. This study had complete baseline information (Q1) on all subjects and analysis revealed that non-respondents did not differ significantly in the majority of variables compared to respondents regarding baseline data. Respondents were significantly older compared to non-respondents, and this is consistent with research showing that respondents are usually older compared to non-respondents [56]. Respondents also had statistically significantly fewer children than non-respondents at Q1 indicating that they may feel that they had more time available to fill out the questionnaire. Furthermore, data collected after delivery in 2002 have been deemed to be representative of Swedish women with PPGP [14] and questions included in Q4 were very similar to those in Q1. Although a convenience sample, by using the doublet questionnaires (*n* = 37) we were able to show that most questions showed adequate to excellent agreement in most questions even though the time gap was over 6 months. Nonetheless, the results would have been more reliable if proper test-retest reliability had been performed.

Women in Sweden under the age of 65 are overrepresented in the statistics regarding chronic LBP [58] and the prevalence of LBP in women in the age group 40–49 is estimated to be 35% and is thus higher than the prevalence of PPGP in this study [59]. Before the introduction of international definitions of PGP [6], PGP was defined and confirmed by self-rated pain locations alone or together with clinical tests [21, 60, 61]. Previous questionnaires (Q1-Q3) of the same cohort, this survey used pain drawings to indicate the area of pain in the lumbopelvic area. Consequently, we cannot exclude that some respondents may have been misclassified as PGP often correlates with the same anatomical location as of non-specific LBP. Additionally, there appear to be an increased risk of PPGP in women experiencing both PGP and LBP during pregnancy [3]. Therefore, there is a possibility of misclassification of ‘non-cases’ due to an underestimation of association.

Even though several risk factors were identified concerning PPGP in this study we were not able to demonstrate statistical significance for the predictor variables ‘low back pain before pregnancy in 2002’, ‘days on sick leave due to PPGP/LBP the past 12 months’, and ‘disability pension’. The non-statistical significance for ‘days on sick leave due to PPGP/LBP the past 12 months’, and ‘disability pension’ can most likely be attributed to limited statistical power increasing the risk of type II error. However, the reason why ‘low back pain before pregnancy in 2002’ did not reach significance can only be speculated. One reason could be that persistence and/or duration of pain symptoms may be of more importance in the prediction of PPGP in long-term studies such as this one.

## Conclusion

This long-term follow-up study is unique in its kind demonstrating that full and spontaneous recovery of PGP symptoms, developed during pregnancy, seems to be an unlikely clinical course for a subgroup of women, even 12 years postpartum. Instead, some women appear to transition into chronicity with associated widespread pain problems that may result in long-term sick leave and disability pension. Persistence and/or duration of pain symptoms as well as wide spread pain appears to be the strongest predictors of poor outcome in women with PPGP 12 years postpartum. Therefore, a better understanding of risk factors involved in the development of PPGP is needed to enable the development of a screening tool to identify women at risk. More research is also needed in regard to prevention as well as effective treatment interventions for PGP/PPGP.

## Abbreviations

AUDIT: Alcohol Use Disorders Identification Test
BMI: Body Mass Index
CAM: Complementary and alternative medicine
CI: Confidence interval
IC: Informed consent
IQR: Interquartile range
LBP: Low back pain
MPI-S: Swedish version of the Multidimensional Pain Inventory
NP: Neck pain
NRS: Numeric rating scale
OR: Odds ratio
PGP: Pelvic girdle pain
PPGP: Persistent pelvic girdle pain
Q: Questionnaire
Q1: Questionnaire 1
Q2: Questionnaire 2
Q3: Questionnaire 3
Q4: Questionnaire 4
RMDQ: Roland Morris Disability Questionnaire
SD: Standard deviation
SEK: Swedish krona
SPAR: The Swedish Population Register (Statens Personadressregister)
SRH: Self-rated Health Status
TSP: Thoracic spinal pain
